# Duties of Midwives

**Published:** 1892-10

**Authors:** 


DUTIES OF MIDWIVES, NURSES, AND HEALTH-OFFICERS, AS DEFINED BY THE LEGISLATURE OF RHODE ISLAND.—*Duties of midwife or nurse.*—Section 1. Should any midwife or nurse, or person acting as nurse, having charge of an infant in this State, notice that one or both eyes of such infant are inflamed or reddened at any time within two weeks after its birth, it shall be the duty of such midwife or nurse, or person acting as nurse, so having charge of such infant, to report the fact in writing within six hours to the health-officer, or some qualified practitioner of medicine, of the city or town in which the parents of the infant reside.

*Duties of Health-Officer.*—Section 2. Every health-officer shall furnish a copy of this act to each person who is known to him to act as midwife or nurse in the city or town for which such health-officer is appointed, and the Secretary of State shall cause a sufficient number of copies of this act to be printed, and supply the same to such health-officers on application.

*Penalty.*—Section 3. Every person who shall fail to comply with the provisions of this act shall be fined not exceeding one hundred dollars, or imprisonment not exceeding six months, or both.

Section 4. This act shall take effect, July 1, 1892.—*Ex.*

